# Quantifying cancer cell plasticity with gene regulatory networks and single-cell dynamics

**DOI:** 10.3389/fnetp.2023.1225736

**Published:** 2023-09-04

**Authors:** Sarah M. Groves, Vito Quaranta

**Affiliations:** ^1^ Department of Pharmacology, Vanderbilt University, Nashville, TN, United States; ^2^ Department of Biochemistry, Vanderbilt University, Nashville, TN, United States

**Keywords:** plasticity, gene regulatory networks, dynamical systems, ScRNA-seq, cancer, network physiology

## Abstract

Phenotypic plasticity of cancer cells can lead to complex cell state dynamics during tumor progression and acquired resistance. Highly plastic stem-like states may be inherently drug-resistant. Moreover, cell state dynamics in response to therapy allow a tumor to evade treatment. In both scenarios, quantifying plasticity is essential for identifying high-plasticity states or elucidating transition paths between states. Currently, methods to quantify plasticity tend to focus on 1) quantification of quasi-potential based on the underlying gene regulatory network dynamics of the system; or 2) inference of cell potency based on trajectory inference or lineage tracing in single-cell dynamics. Here, we explore both of these approaches and associated computational tools. We then discuss implications of each approach to plasticity metrics, and relevance to cancer treatment strategies.

## 1 Introduction

### 1.1 Overview

In the field of Network Physiology, cancer systems biology occupies an intriguing position. On the one hand, widespread research efforts advance data production from genes to patients and, in parallel, improving analytical methods for inferring molecular networks from these large datasets are providing insights that both leverage and go beyond reductionism-based knowledge. On the other hand, the built-in plasticity of heterogeneous cell states in tumors and the consequent lack of ground truths typically elucidated in physiological systems create profound uncertainty about structure and dynamics of cancer networks, whether inferred from top-down or bottom-up approaches.

In this review, we place studies on cancer cell plasticity and its underlying network dynamics in the context of broader studies on the regulation of cell plasticity in physiological self-organizing systems, as it occurs in, for example, brain or embryo development (Ivanov, 2021). In a nutshell, cancer has been understood as a disease in which regulation of the cell cycle is lost and cell proliferation has become a runaway process. This is an actionable perspective that has led to many advances in cancer treatment. However, in a larger sense, cancer is a disease of lost cell identity: tumors can be shrunk, slowed down, or almost eradicated, but in the vast majority of cases they relapse in a treatment-resistant or -tolerant state. Our current understanding of relapse is rooted on studies that unambiguously determined the heterogeneous nature of cancer cell states in a tumor. More recently, transitions among these states have been convincingly demonstrated. In this Introductory section, the evidence for tumor heterogeneity and cancer cell plasticity is first summarized, and the case is made for the key role of quantitative metrics for heterogeneity and plasticity.

In the rest of Introduction, theoretical frameworks for plasticity are recalled. The current noisy landscape of information formed by torrents of publications and a dataset tsunami can be overwhelming. We find it essential to grasp for theory as an anchor in reality, and a means for producing knowledge platforms that can be hardened and continuously improved upon (or falsified).

In later sections, current attempts at unveiling the mechanistic basis for plasticity are reviewed, with emphasis on the role of the dynamics of Gene Regulatory Networks (GRNs), and the dynamics of single-cell state transitions. This focus was motivated both by our direct experience in these areas, and by a broadening group of investigators that are collectively producing remarkable advances.

### 1.2 Cell heterogeneity and plasticity in cancer

Heterogeneity within tumors has been shown to be critical for acquired resistance to therapy in many cancer types (Altschuler and Wu, 2010; Calbo et al., 2011; Marusyk et al., 2012; Huang, 2013; Frick et al., 2015; Pisco and Huang, 2015; Jia et al., 2017; Lim et al., 2017; Su et al., 2019; Nath et al., 2021; Yabo et al., 2021). Several layers of heterogeneity exist and play a large role in cancer systems (Elowitz et al., 2002; Brock et al., 2009; Feinberg and Irizarry, 2010; Gupta et al., 2011; Pisco and Huang, 2015; Caiado et al., 2016; Kumar et al., 2019; Hayford et al., 2021). Genetic heterogeneity results from selection of mutants, each of which may respond differently to treatment. Non-genetic (or epigenetic) heterogeneity is dependent on epigenetic regulation of phenotype and can be attributed to several sources, including variability in chromatin accessibility, DNA methylation, and DNA-binding proteins that regulate transcription levels of genes. Finally, stochasticity arises from intrinsic sources, such as the probabilistic nature of biochemical reactions within a cell, or extrinsic sources, such as local fluctuations in chemical concentrations in the microenvironment (Swain et al., 2002). While transient, this variability can probabilistically drive transitions between phenotypes (Feinberg and Irizarry, 2010; Gupta et al., 2011; Liao et al., 2012; Hayford et al., 2021).

**FIGURE 1:**
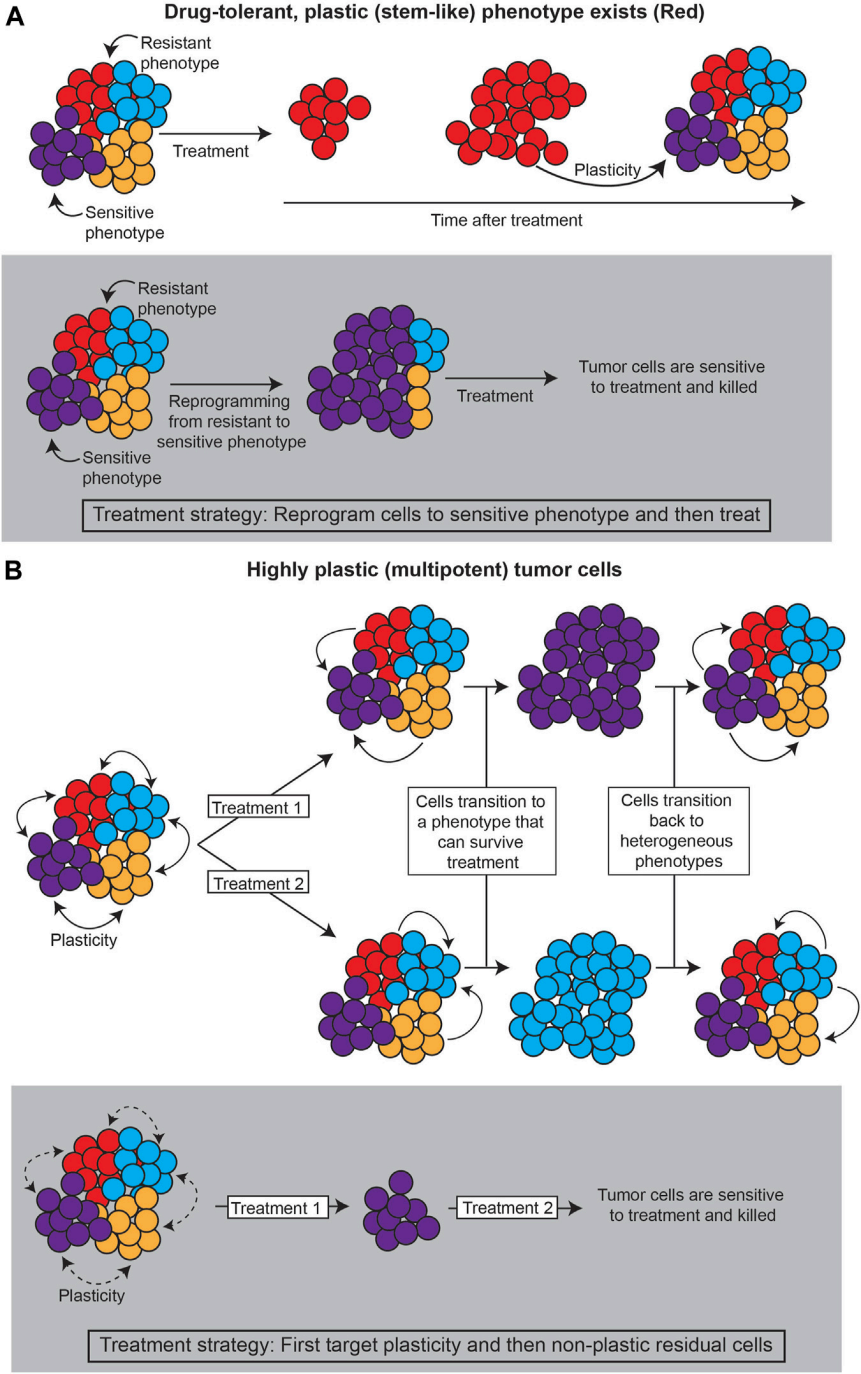
Strategies for treating plastic cancer systems. **(A)** If a specific subpopulation of the tumor is capable of plasticity (such as cancer stem cells), the tumor can be treated by reprogramming the tumor away from this population. **(B)** If the tumor evades treatment through cell state dynamics, plasticity itself must be targeted, such as by decreasing chromatin accessibility that allows cancer cells to change phenotype.

Together, these layers of heterogeneity—genetic, epigenetic, and stochastic—define the variability in phenotype. There is a critical need to quantify these levels of heterogeneity in cancer systems, as distinct phenotypes will presumably respond differentially to treatment, and changes in heterogeneity can lead to acquired resistance (Pisco and Huang, 2015; Brady et al., 2017; Jia et al., 2017; Mu et al., 2017; Zou et al., 2017; Jolly et al., 2018; Risom et al., 2018; Arozarena and Wellbrock, 2019; Su et al., 2019; Nath et al., 2021). Such dynamics of phenotype, or phenotypic plasticity, can lead to differential treatment response and/or resistance in several ways, including: 1) the existence of highly plastic, drug-resistant states; and/or, 2) cell state dynamics that evade treatment (Figure 1).

First, a particular phenotype may be intrinsically less susceptible to treatment, so transitions to this “drug-tolerant persister” phenotype can promote tumor survival (Sharma et al., 2010; Liau et al., 2017; Paudel et al., 2018; Risom et al., 2018; Jia et al., 2020; Cabanos and Hata, 2021; Oren et al., 2021). Often, such a phenotype has stem cell-like properties, suggesting the plastic potential of stem cell-like phenotypes is intrinsically tied to treatment response (Gupta et al., 2011; Chisholm et al., 2015; Liau et al., 2017; Wainwright and Scaffidi, 2017; Smith et al., 2018; Lytle et al., 2019; Neftel et al., 2019; Chan et al., 2021; Yabo et al., 2021). Lineage tracing analyses, as described in Section 3.3., that investigate the underlying mechanisms of the persister state and the transition paths towards it can point to strategies for reprogramming such states to sensitivity.

Second, cell state dynamics between various phenotypes can promote tumor survival by adaptation to treatment (Zhou and Li, 2016; Neftel et al., 2019; Wouters et al., 2020; Gay et al., 2021; Nath et al., 2021; Groves et al., 2022; Sutherland et al., 2022). In these cases, reprogramming cells towards a drug-sensitive state is infeasible, because the high degree of cell state transitions can allow for any cell state to become insensitive again. In this case, it would appear that plasticity itself should be the target.

In both scenarios, it is necessary to quantify the phenotypic plasticity of cancer cell states and the mechanisms underlying cell state dynamics, towards the goal of identifying therapeutic strategies that diminish the plastic capabilities of the tumor as a whole (Huang and Kauffman, 2013). Waddington’s landscape is a useful metaphor for understanding how cancer cells shift between phenotypes and can be quantified through the underlying gene regulatory network dynamics or via statistical mechanical modeling of cell state dynamics, such as those seen in single cell transcriptomics datasets.

### 1.3 Waddington’s landscape in cancer

In 1957, C.H. Waddington proposed the concept of an epigenetic landscape for understanding the regulation of phenotype in the context of biological differentiation (Waddington, 1957). In this analogy, cells roll downhill through canalized channels or “chreods” representing differentiation pathways. Cells at the top of the landscape are pluripotent stem cells, and as they travel down the landscape, they gradually become more committed to a particular cell fate. Thus, the epigenetic landscape could be thought of as a tool to uncover how epigenetic regulation in a cell (e.g., through chromatin accessibility or DNA-binding of transcripton factors) controls the cell’s phenotype. Waddington initially characterized this regulation as a complex system of interactions that he illustrated as strings pulling on and shaping the landscape from below (Waddington, 1957).

**FIGURE 2:**
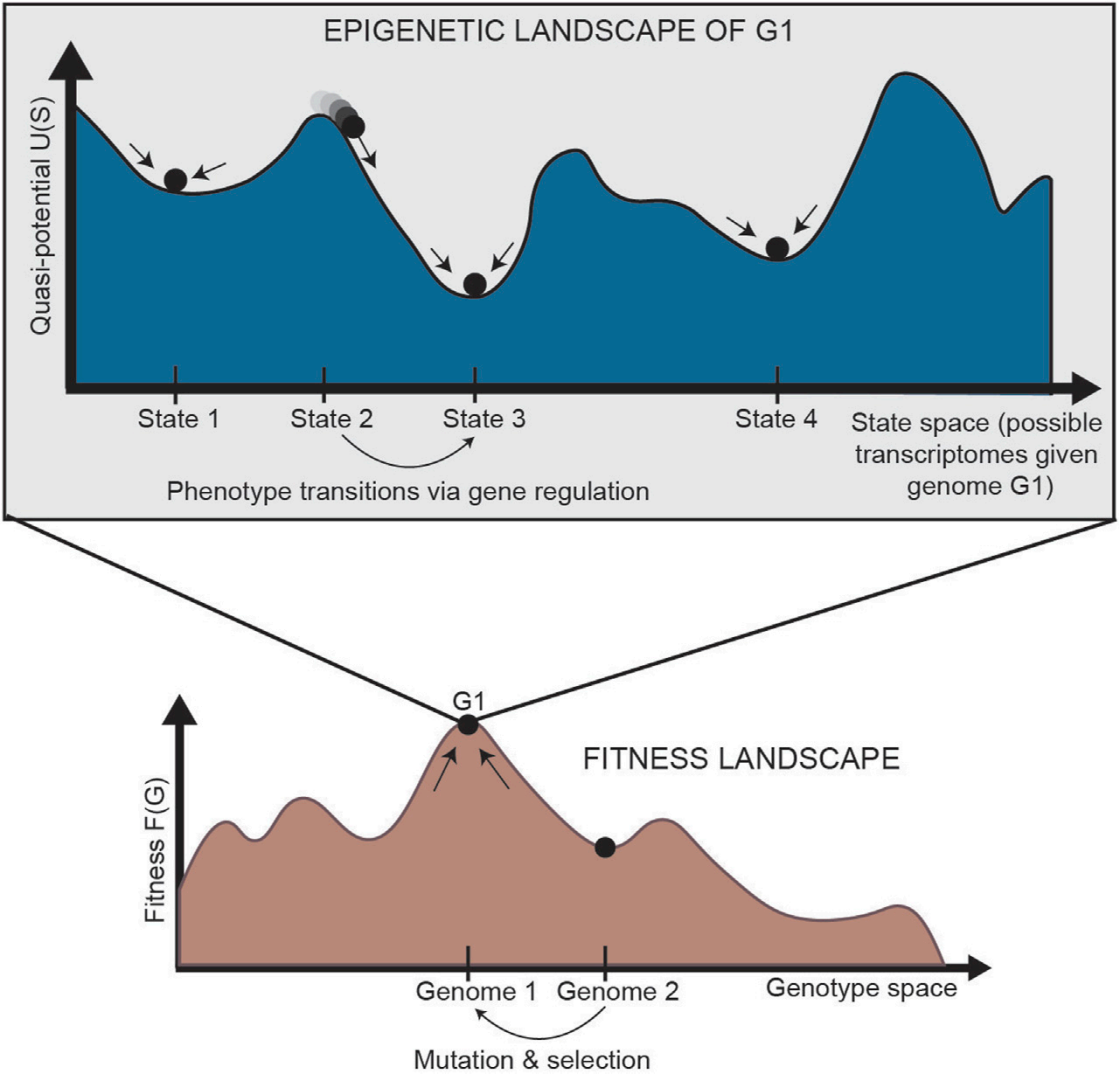
Relationship between the fitness landscape and epigenetic landscapes [adapted from Huang (2013)]. Each epigenetic landscape is associated with a single genome (G1). Selection of high-fitness mutants can be represented by cells “climbing” up a fitness landscape, where each point along the horizontal axis is a different genome. For a specific genome, we can imagine an entire epigenetic landscape that characterizes the phenotypes associated with that genome (since there is not a one-to-one, but one-to-many, relationship between genotype and phenotype). Phenotypic transitions through epigenetic mechanisms allow for movement through the epigenetic landscape. G: genetic state; S: epigenetic state.

In normal development, cells are generally isogenic. In cancer, however, where the mutation rate is higher and multiple subclones may exist within a single tumor, genetic heterogeneity can be represented by a “fitness landscape” (Figure 2, bottom). In this landscape, mutants with higher fitness will be selected for via Darwinian evolution. For each location in the fitness landscape (each genome), an entire Waddington landscape of phenotypes exists (Figure 2, top). Similar to Waddington’s original conception, cells in the epigenetic landscape “fall downhill” towards the states with the lowest “potential.” These phenotypic transitions depend on the instability of each cell state, and a cell’s ability to transition can be defined by its plasticity.

### 1.4 System attractors, instability, and plasticity

**FIGURE 3:**
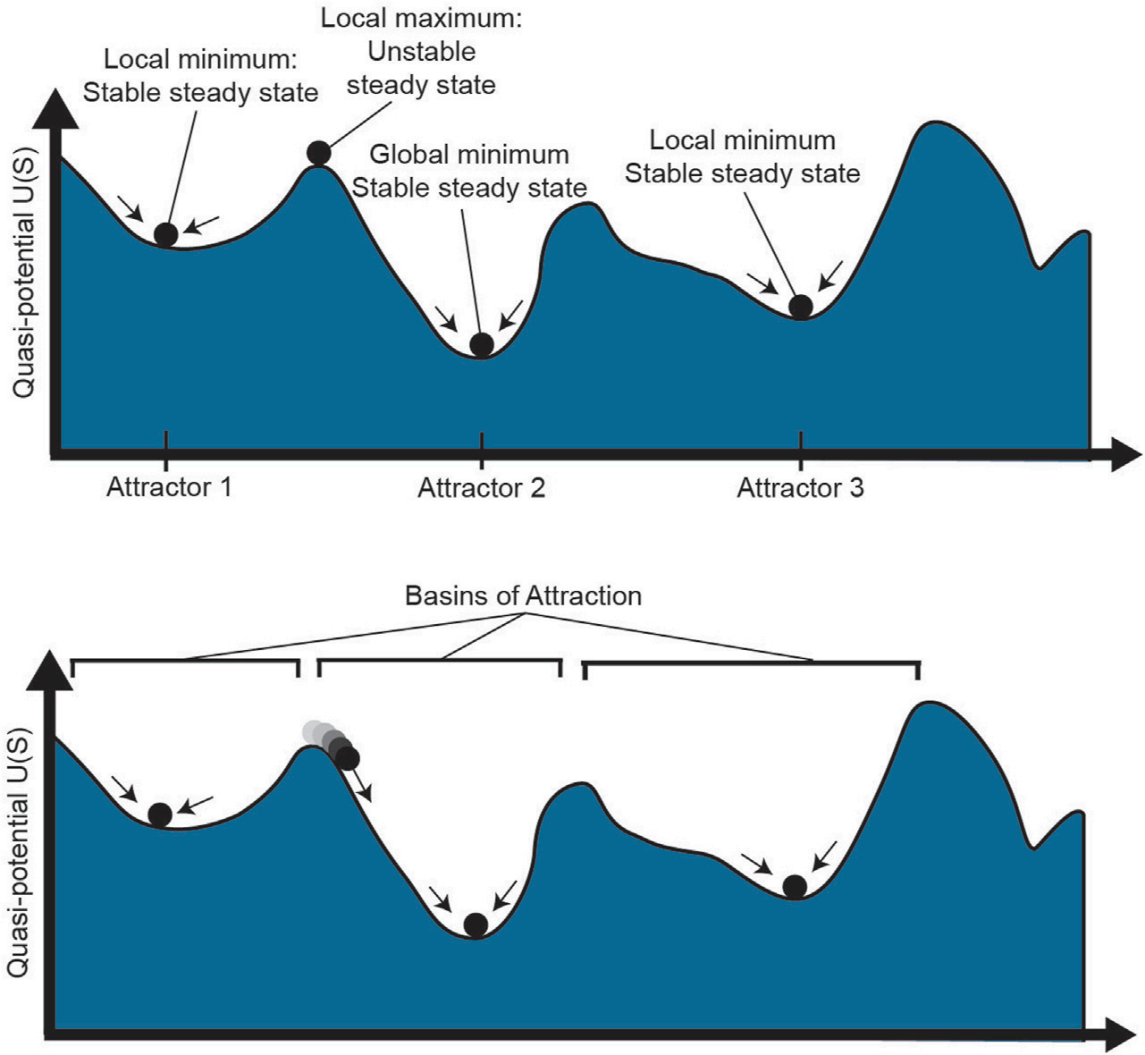
Phenotype stability and attractors. The epigenetic landscape shown above has multiple stable and unstable steady states. While a cell at a local maximum could technically be a steady state, small stochastic perturbations to the cell will quickly push it one direction or another towards a local minimum. Attractor 2 has the lowest potential as the global minimum. The region around each attractor where cells will move towards the attractor is known as the basin of that steady state. S: epigenetic state.

The notion of plasticity goes hand in hand with the dynamical systems theoretical concept of instability. In dynamical systems, stability of a state requires more than stationarity; a stable state is one that is resilient to perturbations such that, after external influences such as changing microenvironmental conditions, the system returns to its original state (Huang, 2013). This idea is represented in the potential landscape, in which cells roll downhill toward local minima, as shown in Figure 3 (top). While there may be steady states throughout the landscape, such as the top of a flat hill or the bottom of a valley, a small push to a cell on top of a hill will cause it to roll down to a local minimum, far from its original starting state. On the contrary, a cell in a local minimum is resilient to small perturbations: it is in a stable “attractor” state of the landscape (Huang, 2009). The high-dimensional region around the attractor where a cell will roll towards the attractor is called the basin of attraction (Figure 3, bottom). Cell states with larger basins of attraction can withstand larger perturbations to their cell state, thereby demonstrating resilience of the system.

In dynamical systems theory, plasticity is a weaker kind of stability, in which a perturbed system neither returns to its original state nor escapes from it, but instead tracks the environmental change (Huang, 2013). However, in biology, plasticity and instability are often thought of as interchangeable: a more plastic cell state responds to an external perturbation by changing its state to a larger degree. In this view, cells with higher potential on the landscape are considered more plastic, as they are more likely to move through the landscape towards a lower-potential attractor. Quantifying both the quasi-potential of the landscape and potential trajectories through a landscape can elucidate the role of phenotype plasticity in cancer, such as plasticity in response to treatment. For example, by quantifying quasi-potential, one can identify highly plastic and/or stem-like cells that may be responsible for tumor propagation (Sharma et al., 2010; Grosse-Wilde et al., 2015; Chan et al., 2021; Gay et al., 2021). By identifying trajectories through the landscape of a tumor cell population, one can characterize the paths cells take epigenetically during tumor development, tumor metastasis, persistence and acquired resistance in response to treatment.

### 1.5 Quantifying plasticity as quasi-potential of Waddington’s landscape

While Waddington intended this picture purely as a metaphor, it has now been quantified in various ways, borrowing ideas from physics and dynamical systems theory to describe the underlying regulation of these processes (Wang et al., 2008; 2011; Zhou et al., 2012). The height of the landscape describes instability of each phenotype as a “quasi-potential,” a correlate of gravitational potential in a physical landscape (Figure 2, top). Quantification of this quasi-potential is informative for processes in which plasticity and instability plays a central role, including cancer systems (Huang et al., 2009; Huang and Kauffman, 2013; Hanahan, 2022). By modeling potential in an epigenetic landscape of phenotypically heterogeneous populations, one can better determine ways to control the permissivity of phenotype and prevent reprogramming of cell identity from a sensitive phenotype to an insensitive one, as often seen in acquired resistance.

Borrowing from physics, movement of cells in the landscape (i.e., changes in **x**_**i**_ (x_1_,x_2_, … ,x_N_) over time, where x is the location of a cell in phenotype space) may be due to some “force” **F**(**x**), similar to the effect of gravity on movement through a physical landscape. A potential, **U**(**x**), can be defined such that the change in phenotype is equal to the gradient of this potential:

$$\frac{d\vec{x}}{dt}=F\left(\vec{x}\right)=-\nabla U\left(\vec{x}\right)$$

Cells will therefore “roll down” the gradient towards states with lower potential. It is worth noting that most high-dimensional, non-equilibrium biological systems are not simple gradient systems, and therefore the vector field is sometimes decomposed into two components: the gradient of some quasi-potential, and a remainder term (Wang et al., 2008). Still, the gradient term has been successfully used to understand pathways of transition through epigenetic landscapes, describing everything from differentiation to cell fate reprogramming (Wang et al., 2006; 2010; Zhou and Huang, 2010; Zhou et al., 2012; Wu and Wang, 2013a; 2013b; Li and Wang, 2014a; 2014b; Wang, 2015; Zhou and Li, 2016; Luo et al., 2017; Yan et al., 2019). Furthermore, the high dimensionality of complex biological systems can pose a problem for characterizing the structure of an interpretable, lower-dimensional epigenetic landscape. Recent work addressed this problem using a dimension reduction approach of the landscape (DRL), which projects high-dimensional landscapes into a lower-dimensional coordinate system based on variance in an associated probability density function (Kang and Li, 2021). This method was applied to cancer systems in the context of epithelial to mesenchymal transitions and metabolism (Kang and Li, 2021).

Several systems biology approaches have been developed to determine the driving force **F**(**x**) that shapes the epigenetic landscape and defines phenotypic plasticity (Huang, 2012; Zhou et al., 2012; Devaraj and Bose, 2020). Classical dynamical systems modeling of underlying gene regulatory networks is a bottom-up approach that can explain how phenotypic transitions are dependent on regulation of gene expression by transcription factors (TFs) (Bhattacharya et al., 2011; Wang et al., 2011; Joo et al., 2018). Alternatively, phenomenological top-down approaches based on analysis of large ‘omics’ datasets can approximate the potential landscape. For example, single-cell sequencing of transcriptomes samples the density of cells in the landscape and trajectory inference methods uncover transition paths between attractors, i.e., cell states (Saelens et al., 2019). These two orthogonal approaches are detailed in the following two sections.

## 2 Modeling plasticity in epigenetic landscapes via gene regulatory networks

### 2.1 Gene regulatory network structure and dynamics

**FIGURE 4:**
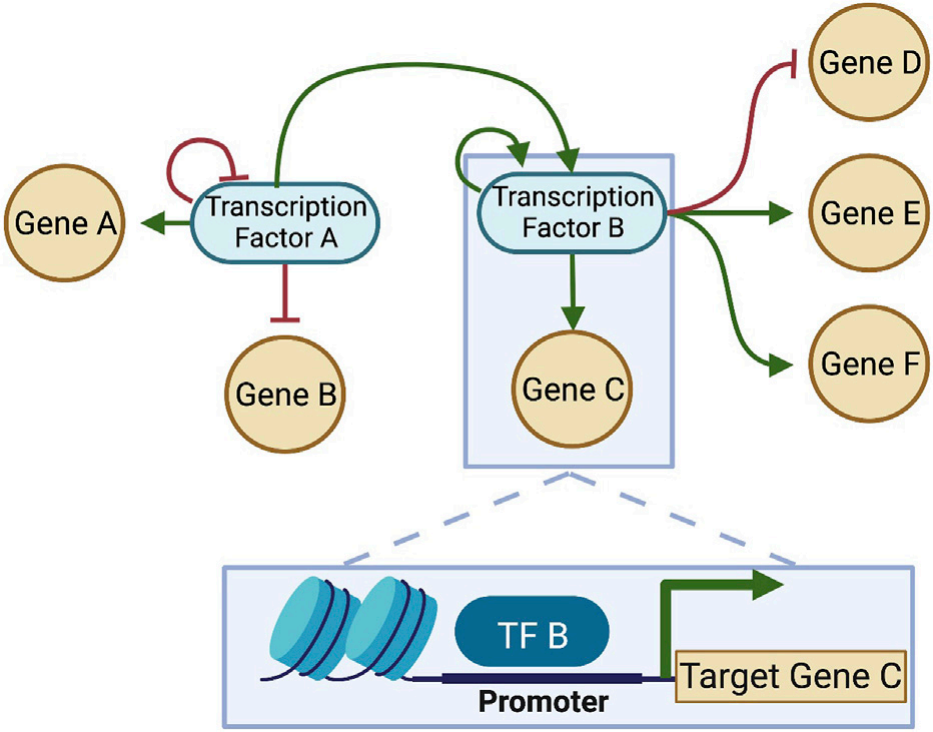
A gene regulatory network (GRN) constructed from interactions between DNA-binding TFs and target genes. Each connection in the GRN represents a physical interaction: the “parent node” is a transcription factor (protein) that binds to the promoter or enhancer region associated with a target gene, which may or may not code for another transcription factor. When the target gene is also a transcription factor, the connection is part of the GRN; otherwise, if the gene does not make a protein that feeds back into the network, it is often pruned, since the transcription and translation of that gene will not affect the network dynamics. Here, two transcription factors that interact are considered, and each regulates itself (shown as feedback loops in the network). Each transcription factor regulates multiple genes. TF-binding for one of the interactions is shown in the box at the bottom of the figure. Green arrows: positive regulation (activation); Red bars: negative regulation (inhibition). Created with BioRender.

**FIGURE 5:**
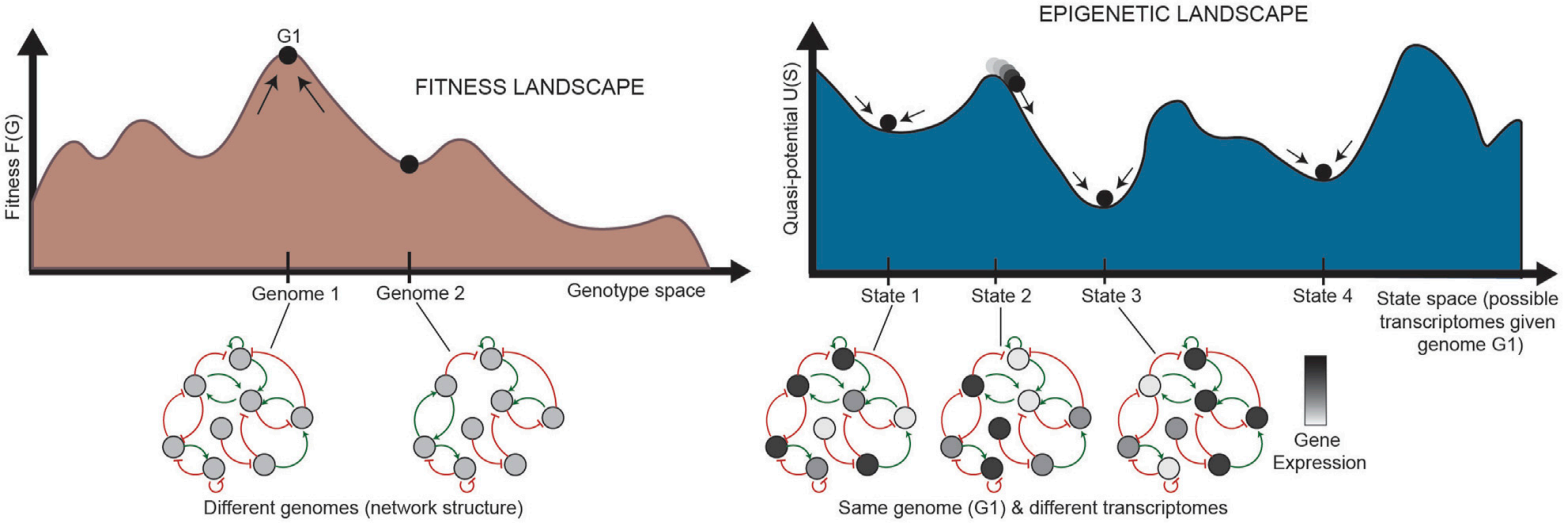
Relationship between landscapes and GRNs (adapted from Huang (2013). (Left) Each state in the fitness landscape (a single genome) is associated with a different GRN structure; mutations can affect the physical interactions between TFs and their target genes, causing the addition or removal of nodes or connections. (Right) Each state in the epigenetic landscape, alternatively, has the same genome, and thus the same structure of a GRN. The states in the landscape here represent different states of the same network, where the same nodes in the network are expressed at different levels. The stability of each pattern of expression partially determines the shape of the landscape. G: genetic state; S: epigenetic state.

To understand the driving force **F**(**x**) that defines the landscape quasi-potential, first it is important to understand gene regulatory networks (GRNs). A GRN is established by the fact that certain genes encode TFs which are capable of binding to DNA and regulate transcription of other genes into RNA. Because TFs can also control the transcription of other TFs (and sometimes themselves), a network of TFs and the genes they regulate can be constructed (Figure 4). The structure of the GRN for a particular cell is hardcore in the genome of a cell, as shown in Figure 5 (left), since each interaction in the network is a molecular interaction between a DNA-binding protein and the cis-regulatory loci (such as promoter and enhancer regions) for a particular gene (Huang, 2013). On the other hand, the dynamics of the network are described by the collective changes in gene expression over time. The dynamics of a GRN allow for various stable states dependent on the expression of genes in the network (Figure 5, right). Therefore, the state of the GRN, given by the expression levels of the genes within it, maps to a single location on the epigenetic landscape—the phenotypic state.

Quantifying the dynamics of TF binding can be calculated by adapting Hill kinetics to describe the rate at which a target gene is transcribed when regulated by TFs (Hill, 1913). The Hill equation is a sigmoidal function that describes activation (or repression) of a gene as dependent on the concentration of a regulator until it reaches saturation. This is a relatively realistic description of many gene control functions and can be derived directly from the binding of the TF to the promoter site. The dynamics, or the change over time, of each TF in the network can therefore be represented as a function of all “upstream” parent nodes in the network that influence its transcription. The system of such differential equations, where each TF in the network has a corresponding equation for its rate of change, defines the complete dynamics of the system. Based on this system of equations, GRN dynamics are equivalent to the driving force that pushes cells down the gradient of the potential in the landscape.

Several researchers have directly solved such systems of ODEs to quantify the plasticity of various systems (Wang et al., 2010; Wang et al., 2011; Zhou et al., 2012; Li and Wang, 2014b; Zhou and Li, 2016; Devaraj and Bose, 2020). Transition paths between stable states can then be calculated, such as by using a path-integral approach (Li et al., 2016; Lang et al., 2021). However, for high-dimensional systems, this system of equations often becomes intractable. Instead, several computation methods have been developed to approximate network interactions.

### 2.2 GRN simulations can model phenotypic transitions

In 1969, Stuart Kauffman introduced the idea of Boolean network models for biological systems (Kauffman S., 1969; Kauffman, S. A. 1969). Kauffman posited that, “while finely-graded intermediate levels of gene activity could occur,” genes tended to be very active or very inactive (Kauffman, 1971), consistent with switch-like Hill kinetics with a high Hill coefficient. Therefore, it is often useful to idealize the control of gene expression as a binary switch. Boolean logic determines the activity level of each gene given the binary states of its upstream regulating TFs by approximating the Hill equation, turning the smooth, monotonic function into a step function with activation (or repression) threshold of S (Kauffman, 1971; Glass and Kauffman, 1973; Thieffry and Thomas, 1998). Since Kauffman’s original idea, several studies have shown the utility of conceptualizing gene regulation as a set of binary genes coupled together through Boolean functions (Albert et al., 2008; Pomerance et al., 2009; Saadatpour and Albert, 2013; Masoudi-Nejad et al., 2015; Steinway et al., 2015; Zhou et al., 2016; Correia et al., 2018; Joo et al., 2018; Yachie-Kinoshita et al., 2018; Wooten et al., 2019).

While a Boolean approximation for transcriptional regulation is realistic for many biological systems, some genes are regulated by multiple TFs in a manner that does not use Boolean logic. For example, Kalir and Alon (2004) showed that gene regulation in an *E. coli* network of flagella biosynthesis follows a summation function (SUM), rather than Boolean logic gates (AND, OR, and NOT). Several studies have shown other functions, including complex functions with many inputs, are also possible (Yuh et al., 1998; Beer and Tavazoie, 2004; Istrail and Davidson, 2005). To model such complex systems, other types of networks must be used. One such approach is to adapt Boolean networks using probabilistic rules (Dorigo, 1994; Shmulevich et al., 2002; Li et al., 2007; Trairatphisan et al., 2013; Tercan et al., 2022). Probabilistic Boolean Networks (PBNs) find a set of functions for each node in the network, each with an associated probability of predicting the target node.

In order to understand systems with non-Boolean gene regulatory functions, other probabilistic methods of network inference, known as probabilistic graphical models (PGMs), may be used (Wang et al., 2005; Zou and Conzen, 2005; Li et al., 2011; Baba et al., 2014; Sanchez-Castillo et al., 2017). These models have multiple advantages over Boolean approaches. For example, they can infer non-linear relationships between TFs, so that the rule of interaction is not required *a priori* to have a particular form such as a Boolean function. One such PGM, known as a Bayesian network, considers a GRN to be a network (or graph) where each directed edge represents the probabilistic dependence among genes. PGMs are more phenomenological than Hill kinetics or Boolean network modeling, but they can mine information from transcriptomic data—for example, RNA-seq profiles for the TFs in the network—without assumptions, such as binariness, about the relationships between TFs (Li et al., 2007; Chai et al., 2014).

Several methods focus on predicting network structure alone (Margolin et al., 2004; Langfelder and Horvath, 2008; Huynh-Thu et al., 2010; Aibar et al., 2017; Chan et al., 2017; Moerman et al., 2018). Some of these approaches utilize similarity metrics on transcriptomic data, e.g., to identify co-expressed gene modules or find relationships between genes with high mutual information, such as WGCNA, GENIE3, ARACNE, or PIDC (Margolin et al., 2004; Langfelder and Horvath, 2008; Huynh-Thu et al., 2010; Chan et al., 2017). While these approaches have been successfully applied to several systems, including cancer, they can often find spurious relationships that do not correspond to physical interactions (cis-regulatory motifs, such as transcription factors binding to the promoter of a target gene). More recent methods can also incorporate this binding information to predict regulatory relationships. For example, SCENIC builds a network structure based on gene co-expression modules and transcription factor binding motif information from the RcisTarget database (Aibar et al., 2017). These tools identify network interactions that coordinate changes in cell identity, but do not predict single cell dynamics.

Many computational algorithms have also been developed to infer both network structure and single-cell dynamics based on Boolean, Bayesian, or other regulatory rules (Chan et al., 2017; Khan et al., 2017; Sanchez-Castillo et al., 2017; Chen and March 2018; Castro et al., 2019; Dunn et al., 2019; Wooten et al., 2019; Aalto et al., 2020; de Sande et al., 2020; Pratapa et al., 2020; Ramirez et al., 2020; Su et al., 2022; Hérault et al., 2023; Kamimoto et al., 2023). For example, BooleaBayes uses probabilistic Boolean rules to predict master regulators of heterogeneous phenotypes that, when perturbed, could destabilize particular phenotypes and therefore change the phenotypic composition of a tumor (Wooten et al., 2019; Olsen et al., 2021; Groves et al., 2022). SCODE models a GRN via ODEs using the gene expression matrix and associated pseudotime from a single-cell dataset and uses this GRN to reconstruct the expression dynamics (Matsumoto et al., 2017). CellOracle uses scRNA-seq and scATAC-seq to generate GRNs and simulate changes in gene expression following experimental perturbations (Kamimoto et al., 2023).

Regardless of the limitations or assumptions of network inference algorithms, these methods require biological data to fully characterize a system. Transcriptomics data are often used, sometimes in combination with other types of epigenomic or proteomic information (Margolin et al., 2004; Langfelder and Horvath, 2008; Liu et al., 2016; Duren et al., 2017; Ramirez et al., 2017; Wooten et al., 2019). Today, single-cell RNA-sequencing (scRNA-seq) is commonly used to obtain a more granular picture of transcriptional regulation and stable phenotypes in a system than bulk sequencing data can provide. Top-down, phenomenological approaches for modeling the epigenetic landscape can also utilize scRNA-seq data directly to find empirical patterns of expression. Because intratumoral heterogeneity and plasticity are relevant to acquire resistance in cancer, it is important to determine how cells change in phenotype in various contexts. These top-down approaches work towards the long-term goal of personalizing treatment by providing a framework for understanding plasticity in an individual patient’s tumor.

## 3 Modeling plasticity in epigenetic landscapes via single-cell dynamics

Single-cell sequencing methods have paved the way for data-driven approaches to quantifying plasticity. While classical dynamical systems modeling—i.e., modeling a GRN that determines a quasi-potential landscape—has the advantage of being predictive, it can be difficult or impossible to model the complete dynamics of a high-dimensional system. Alternatively, it is possible to use a data-driven, bottom-up approach by modeling single-cell dynamics as a Markovian process, which can identify transition paths heuristically from scRNA-seq data.

Borrowing once again from physics, a drift-diffusion equation can model the change in cell density for a given region of gene expression space (or, analogically, the phenotypic landscape):

$$\frac{\partial c}{\partial t}=-\nabla \left(cv\right)+Rc$$

where c is cell density of a given region of gene expression space, R describes the rate of accumulation and loss due to cell proliferation, death, and movement through the region, and v is the net average velocity (Weinreb et al., 2018). With additional assumptions, we can model the velocity as related to the deterministic average velocity field (due to the epigenetic landscape, for example,) and a stochastic component related to diffusion. This velocity field may be calculated heuristically from pseudo-temporal information using trajectory inference methods and can predict cell-state transitions in the epigenetic landscape (Qiu et al., 2022). Furthermore, drift-diffusion modeling of cell dynamics along a high-dimensional manifold in gene expression space can be used to infer dynamics through a Markov chain, with defined transition probabilities between cell states (Weinreb et al., 2018).

### 3.1 Trajectory inference and pseudotime as a measure of plasticity

Trajectory inference algorithms also aim to understand changes in cell density by ordering cells along a trajectory based on transcriptomic similarity, empirically determining transition paths in the system (Trapnell et al., 2014; Guo et al., 2016; Haghverdi et al., 2016; Welch et al., 2016; Qiu et al., 2017; Herring et al., 2018; Saelens et al., 2018; Setty et al., 2019; Wolf et al., 2019; Stassen et al., 2021). These trajectory inference algorithms tend to search for an underlying manifold of the data to delineate graph-based trajectories. By interrogating the structure of the single cell data in gene expression space, multifurcations, trees, and other graph structures can be identified. While these methods are unbiased and often unsupervised, they tend to require identification of a “root cell” to determine the directionality of transitions, as multiple trajectories could be explained by the same graph structure. Such a root cell, or source, can be thought of as having a high degree of plasticity, as defined by the quasi-potential of the underlying landscape. Therefore, these methods require *a priori* knowledge of the high-plasticity states of a system but are useful for identifying transition paths from these states.

Some methods utilize time-series data to determine directionality by optimal transport-based algorithms (Kimmel et al., 2019; Schiebinger et al., 2019; Marjanovic et al., 2020). Because scRNA-seq is a destructive method, the same single cell cannot be monitored and sequenced over time. Optimal transport-based methods overcome this experimental constraint by inferring “temporal couplings” across timepoints to determine the most likely phenotypic “descendants” of each cell at later timepoints. Ultimately, lineage tracing provides a benchmark for interrogating trajectories, as cell lineages across timepoints are identified via “barcodes,” thereby linking cell state in early timepoints to cell fate in later timepoints (Griffiths et al., 2018; Wagner and Klein, 2020; Wang et al., 2021).

### 3.2 RNA velocity-based measures of plasticity

**FIGURE 6:**
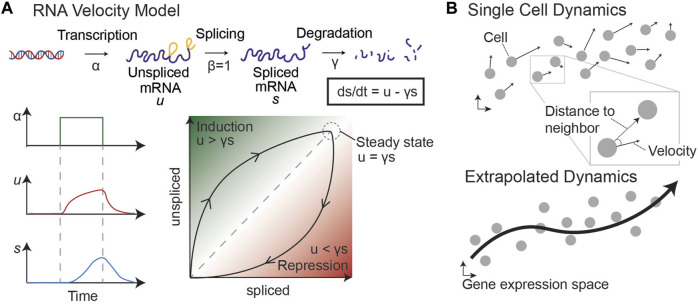
RNA velocity model [adapted from (La Manno et al., 2018)]. **(A)** By modeling transcription, splicing, and degradation of RNA as ODEs, we can determine the steady state proportion of unspliced and spliced RNA and infer dynamics of single cells. An increase in transcription leads to an increase in unspliced and then spliced RNA, with lag time. This difference helps to determine whether a snapshot proportion of unspliced and spliced counts of a particular gene is increasing (induction) or decreasing (repression). **(B)** Velocity vectors in gene expression space are calculated for each individual cell. By comparing each velocity vector to the distance to neighboring sampled cells, we can predict the probability of the cell transitioning to other states (defined by sampled cells). This allows us to generate a Markov chain model and infer dynamics through the single cell data.

In 2018, a novel approach to trajectory inference was developed based on RNA splicing dynamics (La Manno et al., 2018). By fitting an ordinary differential equation (ODE) model of RNA transcription, splicing, and degradation, La Manno et al. discovered that it was possible to infer short-term dynamics on a cell-by-cell basis (Figure 6). RNA velocity infers a steady-state ratio of unspliced to spliced counts of RNA on a gene-by-gene basis to fit the ODE model parameters, such as the degradation rate of the mRNA. As shown in Figure 6, an increase in RNA transcription from a particular gene results in a slow increase of unspliced RNA, followed by a delayed increase in spliced RNA. Therefore, by comparing the unspliced and spliced counts of a gene in each cell in this model, it is possible to determine the future state of each cell.

The timeframe for dynamic predictions is on the order of a few hours, similar to the average splicing rate. However, RNA velocity can be extrapolated to longer timeframes by considering the relationship between a cell’s velocity vector—i.e., the directionality and magnitude of its inferred change in gene expression—and the location of neighboring cells (Figure 6, right). These extrapolated dynamics can be used to make predictions about the future state of cells near the beginning of the trajectory. Because this method does not rely on multiple sampled timepoints or prior knowledge about the “root” cell of a trajectory, it is optimal for understanding the dynamics of systems for which a temporal series of samples is not possible, such as tumor dynamics from single biopsies. Together, these analysis methods can uncover an empirical epigenetic landscape by defining stable phenotypes and transition paths in scRNA-seq data sampled from various cancer systems, including human biopsies, to complement or replace quantification of GRN dynamics.

Since RNA velocity was first introduced, several methods have utilized the approach to quantify plasticity (Bergen et al., 2020; Gorin et al., 2020; Chen et al., 2022; Groves et al., 2022; Lange et al., 2022; Qiu et al., 2022). As described in the original paper, trajectory inference from RNA velocity translates the velocity vectors into a transition probability matrix, which can be used in a Markov Chain model of cell state dynamics (La Manno et al., 2018; Bergen et al., 2020). This approach assumes that movement of cells through a phenotypic landscape is a Markovian process in which cell fate depends only on the current state of the cell. With this assumption, we (Groves et al., 2022) and others (Weinreb et al., 2018; Bergen et al., 2020) proposed to quantify plasticity as a cell’s potential to move towards one or more attractors of the system, i.e., a cell’s ability to traverse a phenotypic landscape. Our metric, termed Cell Transport Potential (CTrP), quantifies the average distance traveled for each cell through a Markovian state transition graph, accounting for multiple possible cell fates (or absorbing states) (Groves et al., 2022). The gain with CTrP is an intuitive connection between cell landscape dynamics and transcriptomics: the higher the CTrP of a cell in a landscape, the larger the mobility in gene expression space expected for that cell. With CTrP, we identified highly plastic cell states across several small cell lung cancer (SCLC) human and mouse experimental models (Gay et al., 2021; Groves et al., 2022). For instance, in a circulating tumor cell-derived xenograft (CDX) model, we were able to determine that resistant tumors post-treatment originated from a small, high-CTrP cell state that arose after chemotherapy (Gay et al., 2021).

Other approaches have quantified plasticity as multipotency by defining possible cell fates for each cell state using RNA velocity-based transition probabilities. For example, CellRank builds on RNA velocity and trajectory inference models (such as pseudotime) to predict fate potentials given the stochastic nature of fate decisions (Lange et al., 2022). CellRank has been used to predict fate probabilities and reprogramming outcomes in several developmental systems (Hersbach et al., 2022; Lange et al., 2022; Van Bruggen et al., 2022; Bono et al., 2023; Matsushita et al., 2023), whereas applications to cancer systems have mainly focused on trajectory inference for the immune compartment rather than cancer cells (Xue et al., 2022; Friedrich et al., 2023; Jainarayanan et al., 2023). DeepVelo uses neural networks to learn transcriptomic dynamics, building a model that predicts trajectories, driver genes, and the effect of *in silico* perturbation on fate decisions (Chen et al., 2022); however, this approach has not yet been applied to cancer systems.

These approaches have been used to predict perturbations that can affect fate decisions. In cancer, these methods could identify treatment options for perturbing cells away from drug-resistant cell types (Wooten and Quaranta, 2017) or cancer attractors as a whole (Huang and Kauffman, 2013; Li et al., 2016).

### 3.3 Lineage tracing to understand cell state transitions

While trajectory inference of single cell sequencing data can provide high granularity for understanding phenotypic heterogeneity in cancer, such approaches to understand plasticity of cancer cells over time must account for the destructive nature of sequencing. Alternatively, lineage tracing methods have long been used to understand the temporal dynamics of cell state in cancer populations, particularly during tumor initiation and in response to treatment (Chakrabarti et al., 2018; Wagner et al., 2018; Weinreb et al., 2020; Wang et al., 2021; Singh and Saint-Antoine, 2023). Single cell time-lapse microscopy has been used to correlate cell state and fate, suggesting the existence of phenotype switching (Bhola and Simon, 2009; Spencer et al., 2009; Bertaux et al., 2014; Chakrabarti et al., 2018). For example, researchers used time-lapse microscopy to understand variability in the onset of apoptosis, finding that protein state gives rise to transient heritability between mother and daughter cells (Bhola and Simon, 2009; Spencer et al., 2009). This can be modeled mechanistically by considering stochastic fluctuations in protein levels (Bertaux et al., 2014). Together, these results connect cell state (assumed to be identical in sister cells) with cell fate (divergence in apoptotic response), and pave the way for understanding how subpopulations of a single tumor can have such different fates (drug sensitivity *versus* tolerance) in response to a single treatment.

More recently, several groups have used a modified Luria Delbrück fluctuation analysis to determine whether resistance to therapy is heritable or a result of transient reprogramming of phenotype (Shaffer et al., 2017; 2020; Russo et al., 2022; Singh and Saint-Antoine, 2023). For example, Shaffer et al. (2017) tested whether resistance to vemurafenib in BRAF-mutated melanoma was genetically heritable or transient. If the drug resistance was transient and due to epigenetic reprogramming, a Luria Delbrück fluctuation analysis would show a tighter distribution of resistant cell colony sizes, because all cells would be equally likely to form a resistant colony. The hypothesis of a transient pre-resistant state that could epigenetically reprogram to a stably resistant state (i.e., persister state) under drug was indeed supported by the results. This state was further characterized by a distinct transcriptional profile (including high expression of EGFR) and activation of transcription factors (JUN, AP-1, and TEAD). Shaffer et al. (2020) then expanded this work into a broadly applicable method, MemorySeq, that combines Luria-Delbrück fluctuation analysis and population-based RNA sequencing. Similarly, Russo et al. (2022) used fluctuation analysis to investigate drug-induced plasticity of colorectal cancer cells. In this cancer system, cell population dynamics were quantified with a mathematical model of transitions to a persister state, which was consistent with a drug-induced, rather than preexisting, persister state.

Together, these experiments and analyses have shown that drug resistance in cancer can arise from epigenetic reprogramming of transient, pre-resistant states, and that high degrees of transcriptional heterogeneity allow for rare cell populations to become stably resistant through plasticity. Importantly, these transitions to a stably resistant state are drug-induced rather than preexisting, solidifying the connection between treatment and plasticity of cancer cells.

## 4 Conclusion

The success of cancer therapies is often limited by mechanisms of cellular persistence and acquired resistance. Non-genetic plasticity has emerged as a major cause of treatment insensitivity or acquired resistance in several cancer types (Marjanovic et al., 2013; 2020; Pisco et al., 2013; Mu et al., 2017; Su et al., 2017; Zou et al., 2017; Qin et al., 2020; Quintanal-Villalonga et al., 2020; Chan et al., 2021; Hanahan, 2022). Targeting plasticity directly has been suggested as a possible treatment option for several cancers, including melanoma, breast cancer, and prostate cancer (Sáez-Ayala et al., 2013; Kemper et al., 2014; Ahmed and Haass, 2018; Risom et al., 2018; Arozarena and Wellbrock, 2019; Chapman et al., 2019; Boumahdi and de Sauvage, 2020; Yabo et al., 2021).

A few different methods for targeting plasticity can be envisioned. First, cell plasticity could be used advantageously to reprogram cells towards more drug-sensitive states (Yuan et al., 2019). For example, master TFs, identified through GRN analyses, could be controlled to direct phenotype switching to attractors that better respond to treatment, as shown in melanoma (Sáez-Ayala et al., 2013).

Second, preventing phenotype switching may be more desirable (Boumahdi and de Sauvage, 2020). Phenotypic plasticity is intrinsic to the epigenetic landscape: By shaping the landscape, GRN dynamics form transition paths and unused attractors, and cells may enter transition paths between stable attractors due to extrinsic perturbations or intrinsic stochasticity (Huang, 2013). The barrier to exit attractors may be lower in cancer than normal cells, with “de-canalized,” shallow valleys and attractor basins enabling cancer cells to stochastically sample the landscape and find new attractors that evade treatment (Jia et al., 2017). Targeting the mechanisms that allow for this stochastic search of drug-tolerant states in the landscape may lower plasticity and acquired resistance to therapy. For example, chromatin remodeling may be a key mechanism by which cells reprogram to other fates, as open chromatin has been shown to correlate with plasticity (Meshorer and Misteli, 2006; Giadrossi et al., 2007; Gaspar-Maia et al., 2011; Burdziak et al., 2023). In fact, a recent study on pancreatic ductal adenocarcinoma used this connection between plastic cells and accessible chromatin landscape to quantify plasticity as the entropy in prediction of transcriptomic fate based on chromatin accessibility (epigenomic state) (Burdziak et al., 2023). In cancers where plastic states with open chromatin landscapes exist, promoting repressive chromatin organization may be able to keep cells from transitioning between phenotypes during tumor progression or treatment evasion.

Modeling this plasticity through GRNs or single-cell dynamics can lead to new approaches to therapy. Development of strategies that target plasticity and systematically reprogram cell identity may ultimately enable to overcome persistence and acquired resistance in cancer. These goals should not be elusive, if they are rooted in our current understanding of mechanisms of GRN regulation. In a sense, it could be useful to start viewing cancer cells as driven by dysregulated GRNs, rather than by some mysterious “malignant” property (i.e., by a misguided absolute priority for self-preservation, as an invading virus or bacteria would do). Such perspective may engender a longer but perhaps more rewarding path to achieving a solution to this devastating disease.

Single-cell state transitions in response to perturbations are also broadly observed in other physiological systems. In fact, perfect adaptation is well-known in unicellular organisms, and adaptability is essential for tissue homeostasis. Thus, reproducible dynamics in physiological platforms, either spontaneous or under perturbation, can be used to place boundaries on cancer adaptive networks. Vice versa, cancer network studies can provide insights into the potential of physiological networks, e.g., in the context of evolution. We submit that bridging these areas of research will eventually broaden perspectives on network physiology.
